# Economic impact of antimicrobial resistance.

**DOI:** 10.3201/eid0702.010228

**Published:** 2001

**Authors:** J E McGowan

**Affiliations:** Emory University School of Medicine, Atlanta, Georgia, USA. jmcgowa@sph.emory.edu

## Abstract

One reason antimicrobial-drug resistance is of concern is its economic impact on physicians, patients, health-care administrators, pharmaceutical producers, and the public. Measurement of cost and economic impact of programs to minimize antimicrobial-drug resistance is imprecise and incomplete. Studies to describe and evaluate the problem will have to employ new methods and be of large scale to produce information that is broadly applicable.


### Economic Impact of Antimicrobial Resistance

**John E. McGowan, Jr.**
Emory University School of Medicine, Atlanta, Georgia, USA

One reason antimicrobial-drug resistance is of concern is its economic impact on physicians, patients, health-care administrators, pharmaceutical producers, and the public. Measurement of cost and economic impact of programs to minimize antimicrobial-drug resistance is imprecise and incomplete. Studies to describe and evaluate the problem will have to employ new methods and be of large scale to produce information that is broadly applicable.

One reason antimicrobial-drug resistance has recently become a concern is its economic impact. The Institute of Medicine estimates the annual cost of infections caused by antibiotic-resistant bacteria to be U.S.$4 to $5 million (1). However, methods for measuring economic impact of resistance are in their infancy, and the studies leave many questions unanswered (2). In this review, I examine perspectives from which economic impact of resistance is important, assess available data about economic methods used for evaluating economic effect, and suggest issues important for these assessments, as well as approaches for further study.

### Economic Impact: Differing Viewpoints

Several viewpoints toward antimicrobial-drug resistance and its impact include those of physicians, patients, health-care businesses, the drug industry, and the public (Table 1).

### Physicians

The view most considered in day-to-day medical care is that of the practicing physician. Physicians focus on individual patients and are motivated by professionalism that demands they seek the absence of disease, most often in persons who are ill when they visit a physician. Thus, the main economic problems that resistance presents for physicians are related to ineffective treatment (e.g., consequences arising from patient death, disease). From this treatment perspective, a production model of the type presented by Scott (3) would relate the existence of multiple antimicrobial agents to likely effectiveness in curing a given patient's infection. To clinicians treating individual patients, availability of more antimicrobial agents than needed would be of little or no concern. However, clinicians would be alarmed by absence of effective agents (the "postantibiotic era" cited frequently since Cohen's publication of that title [4] in 1992). From this viewpoint, the economic impact of diminishing effectiveness of a given drug or group of drugs depends on the availability of other drugs.

### Patients

Patients with infections are likely to have a view similar to that of the physician (Table 1), except that their motivation for participating in the treatment process is their own well-being. Economic impact is also measured in terms of consequences arising from illness and death, specifically the added cost of treatment of a resistant organism, since patients pay retail prices for drugs and services. Such charges are assumed directly when patients pay their own bills or absorbed indirectly when added costs of multiple drugs and services result in increasing premiums for patients who have health-care coverage.

Table 1. Perspectives of economic impact of antimicrobial-drug resistance[a]

| | Focus | Outcome | Time | Motivation | Approach |
|---|---|---|---|---|---|
| Physician | Individual | Health | Short | Professionalism | Treatment |
| Patient | Individual | Health | Short | Health | Treatment |
| Provider | Care group | Lower cost | Short | Profit | Cost containment |
| Industry | Clients | Sales | Short, long | Profit | New drugs, viable old drugs |
| Public | Population | Health | Long | Social good | Lower chance of resistance |

[a]Cordell RL, Solomon SL, Scott RD, McGowan JE Jr, unpub. data.

### Health-Care Businesses

Today, health-care system financial resources in the United States are less frequently controlled by doctors and nurses and more often by administrators, financial managers, third-party payers, and politicians. These people see reduced illness and death as a reasonable goal, but also seek objective evidence that this goal is achieved with fiscal efficiency (i.e., by the least expenditure of increasingly scarce financial resources [5]). Antimicrobial drugs represent a way to provide cost-effective care to patients who are part of a defined population being served. The economic cost of antimicrobial-drug resistance for health-care businesses is in the measures they must take to preserve the effectiveness of antimicrobial agents in the care group. These measures may include costs for a series of different drugs and services, as well as for personnel time, supplies, space, and equipment for institutional programs to deal with antimicrobial-drug resistance (e.g., pharmacy and therapeutics committees,

Address for correspondence: John E. McGowan, Jr., Rollins School of Public Health (Room 442 GCR), Emory University, 1518 Clifton Road, Atlanta, GA 30322; fax: 404-727-8737; e-mail: jmcgowa@sph.emory.edu antimicrobial-drug use review, practice guidelines). The benefit is decreased costs associated with care of patients infected with resistant organisms. Antimicrobial-drug resistance in other settings is of interest to the health business professional only as it affects or has the potential to affect the population receiving the health-care organization's services. From this perspective, health-care organizations may be the easiest setting in which to measure the economic impact of antimicrobial-drug resistance. Here, the analysis is limited to specific antimicrobial drugs, and the impact on care for a specific group of patients can be measured in terms of costs to the specific business. In addition, the costs of measures to preserve effective treatment can also be assessed in relation to other costs.

### Drug Industry

The focus for pharmaceutical firms and other groups providing products for treatment and prevention of infectious diseases (e.g., antimicrobial agents, products to stimulate host defenses, vaccines) is similar to that of the health-care business. This group is also motivated by profit and focuses on potential clients; however, the clients of interest are the potential users of their products--direct (patients) and indirect (health-care systems, governments, and the like)--rather than enrolled subscribers to a health plan. Product sales are the desired outcome, and a short-term view of sales is part of their outlook. However, industry must also take a longer view of the subject and consider the impact of resistance as potential for introduction and sale of new products, necessitating a two-pronged approach. On the one hand, firms wish to maintain the life of their current antimicrobial products, a goal threatened by new patterns of antimicrobial-drug resistance. On the other hand, resistance may make obsolete a competitor's product, opening up the field for a product that may have been less marketable because it cost more or was less safe or effective. In addition, resistance to drugs may produce a niche for a new antimicrobial agent.

### Public ("Societal View")

A final view to be considered is that of public health or the public good. This societal perspective, fueled by the goal of social good, encompasses entire populations, whether of towns, cities, countries, and even the entire world. As the goal here is to maximize health for the whole population, the time frame is usually long term. Since antimicrobial drugs enhance both prevention and treatment of infections, society considers them a valuable resource. As resistance diminishes this resource, a societal goal would be to minimize resistance and therefore the forces that produce resistance.

In the jargon of economics, antimicrobial agents are a scarce resource, that is, one in which consumption (current use) decreases its effectiveness (future value) (6). Any use of antimicrobial agents enhances the likelihood of resistance. From a societal viewpoint, then, appropriate use of antimicrobial drugs for treatment and prevention of infection would lead to an appropriate or acceptable decrease in the value of antimicrobial effectiveness. Conversely, overuse or misuse of antimicrobial drugs would create an inappropriate decrease in these resources. When treating one person leads to decreased effectiveness in treating the next person receiving the drug, society is affected adversely. This impact is often ignored because the short-term outcome and cost of drugs (for example, for perioperative prophylaxis) can be measured readily, and the detrimental effect on long-term usefulness is unquantified for most situations (7).

### Whose Perspective?

The economic costs and benefits of programs to preserve antimicrobial effectiveness must be interpreted in the context of these differing points of view. In any single study, it is essential to keep the same perspective, whichever it may be. Analyses that mix the different points of view in assessment tend to confuse rather than clarify the problem and its extent. For example, the business viewpoint might value loss of effectiveness of a cheap antimicrobial agent as important when it leads to use of a more expensive agent for patient care. In contrast, the medical viewpoint might find loss of effectiveness of the cheaper drug of little consequence as long as other effective drugs are available.

Similarly, the value of antimicrobial effectiveness might differ from an economic viewpoint rather than the medical one. For example, from a public health perspective, the use of antimicrobial agents to promote growth in animals would be evaluated by comparing the relative benefit to food production against the potential for decreasing the effectiveness of prevention and treatment of infections in humans. In contrast, the physician's perspective would evaluate the use of antimicrobial agents in animals in terms of its impact on the effectiveness of specific medical therapeutic agents.

A third example of varying perspectives is the use of measures to control the physician's choice of antimicrobial agents. This step may make great sense to hospital or other health-care administrators when it is likely to produce more efficient use of resources. Yet the control measures might be seen as having no value by clinicians who are willing to use any and all resources to cure their patients.

### Assessing the Economic Impact of Resistance

Net economic impact of resistance can be viewed as the attributable cost of treatment of an infection due to a resistant isolate ("treatment cost") minus the cost of preventing such infections ("prevention cost"). Cost analysis should include consideration of all resources affected by illness or intervention (8). Economic impact of antimicrobial-drug resistance includes a wide range of factors important to various viewpoints (Table 2). The difference in this situation is the added cost for each element associated with infection with a resistant organism compared with the cost for the same element if associated with infection caused by a susceptible microbe (Table 2).

Costs for laboratory tests, radiologic studies, bronchoscopies, or other diagnostic procedures are part of diagnostic costs and primarily of concern to the health-care institution when these costs cannot be passed on to the patient or an insurer. The same is true of costs for purchase and administration of antimicrobial drugs and other therapeutic agents. Patients experience both direct costs of health care and indirect costs (e.g., loss of productivity resulting in reduction in income). Other types of indirect costs of antimicrobial-drug resistance are costs to the drug industry resulting from diminishing marketability of their drugs and costs to businesses for loss of workers' productive time. All these factors are part of the economic impact of resistance.

Studies of the economic impact of resistance have not included measurement of most of these variables. They have

Table 2. Elements of the economic impact of antimicrobial-drug resistance, by perspective affected

| Element | Measurement[a] | Perspective affected |
|---|---|---|
| Death | [Costs associated withtreatment failure (R)] - [Costs associated with treatment failure (S)] | Physician, patient, HCB |
| Illness | [Costs associated with pain, suffering, inconvenience (R)] - [Costs associated with pain, suffering, inconvenience (S)] | Physician, patient |
| Care cost | [Charges for care (R)] - [Charges for care (S)] | Patient |
| Care time | [Time devoted to care (R)] - [Time devoted to care (S)] | Physician, HCB |
| | [Length of process (R)] - [Length of process (S)][b] | Patient, society |
| Diagnosis costs | [Costs for diagnosis (R)] - [Costs for diagnosis (S)] | HCB |
| Treatment costs | [Costs for drugs (additional drugs and treatments, more expensive drugs (R)] - [Costs for drugs (S)] | HCB |
| Diminished marketability | [Market for drug use (R)] - [Market for drug use (S)] | Drug industry |
| New markets | [Market for new drug (S)] - [New market for new drug (R)] (replace current market leader; replace inexpensive drug with more expensive drug; provide new product) | Drug industry |
| Impact on non-treated | [Increased resistance (R)] - [Increased resistance (S)] | Society |

[a]R = extent in patients infected with resistant organism; S = extent in patients infected with susceptible organism; HCB = health-care business.
[b]Costs associated with lack of routine functions during infection, including loss of work, quality of life for patient (includes both inpatient and outpatient components); for society, reduction of useful function in workforce.

usually focused on hospital charges and length of stay, features that are objective and relatively easy to collect compared with other aspects of impact. Recent studies of impact have also included estimates of increased hospital or other institutional stay, incremental specific treatments, and additional diagnostic tests needed for a patient infected with a resistant organism compared with a patient infected with a strain of the same organism that is drug susceptible (Table 3) (9-23). Attempts have also been made to measure death and illness associated with resistant infections. Although these are objective indicators of economic impact, until recently it was impractical to obtain this information on the small patient groups studied at individual hospitals or other single health-care settings. In addition, few studies have been published on the impact of antimicrobial-drug resistance outside health-care locations. Further attention is needed to the community setting, where much of antimicrobial treatment is given and received (24).

Generalizations from single-center studies are hindered by differences in local practices. For example, some centers experience delays in transferring patients with positive cultures for vancomycin-resistant enterococci or methicillin-resistant *Staphylococcus aureus* (MRSA) from acute-care centers to long-term care facilities (25). Estimates of incremental increase in length of hospital stay for these institutions might differ from those where such problems do not exist. Thus, multicenter studies would be needed to obtain data that could be used to generalize about regional or national estimates of impact.

Determining the economic impact of antimicrobial-drug resistance to a given drug may have several facets (26). The relative benefit of being able to use a given drug in comparison with alternatives when this drug is not available must be assessed. Thus, to decide the worth of an antimicrobial drug, several elements must be considered. The incremental cost of treating the patient with alternative agents must be assessed, often by studies in which costs for care of patients infected with isolates resistant to a commonly used agent

Table 3. Examples of studies of economic impact of resistance published in 1999-2000

| Year | First author (ref.) | Study methods | Features measured |
|---|---|---|---|
| 2000 | Soriano (9) | Case-control, cohort | Death, length of hospital stay |
| 2000 | Roghmann (10) | Cohort | Mortality rates at 7 & 30 days, length of hospital stay, direct health-care costs |
| 2000 | Vanhems (11) | Cohort | Death |
| 2000 | Simor (12) | Comparison of cases with arbitrary criteria | Incremental length of hospital stay |
| 2000 | Harthug (13) | Case-control | Death |
| 2000 | Bhavnani (14) | Case-control | Death |
| 2000 | Feikin (15) | Cohort | Death |
| 2000 | Garbutt (16) | Retrospective cohort | Death |
| 1999 | Carmeli (17) | Cohort | Death, length of hospital stay, hospital charges |
| 1999 | Rubin (18) | Modeling, assumption and extrapolation from case reports | Death, direct medical costs |
| 1999 | Weingarten (19) | Case-control | Use of ventilators, length of hospital stay, duration and number of antimicrobial agents, hospital and pharmacy charges |
| 1999 | Gonzalez (20) | Cohort | Death |
| 1999 | Abramson (21) | Case-control | Length of hospital stay, attributable median total cost |

(drug X) are compared with costs for care of patients with isolates that are susceptible to drug X. A potential problem with this type of comparison is that a uniform reference group is not readily available. For example, a study may compare costs for care of patients with susceptible isolates treated with drug X to costs for patients infected with isolates resistant to drug X who are then treated with one or more alternative drugs (e.g., Y,Z), when choice of drug is left to the patient's physician. However, other factors (such as altered renal function or a patient's inability to take oral medications) leading to use of drugs Y or Z to treat patients infected with resistant organisms may also have led to treatment with one of these drugs in patients infected with susceptible organisms. Thus, costs must be evaluated carefully to compare these two groups of patients and account for other factors affecting therapy. Study design may also influence the measured impact of resistance (27,28).

### Current Situation

For these and other reasons, measurement of the economic impact of resistance is imprecise and incomplete. Neither methods for direct measurement nor appropriate surrogate variables have been found for some important features. Methods used have primarily focused on case-control strategies, which have limitations (27).

Further work needed on this aspect of the question includes defining optimal methods of measurement, including more aspects of economic impact, and disclosing the perspective from which the assessment is being made. Measurement of impact of resistance on patients through cost-utility analysis may be helpful as well (29).

### Measuring Benefit of Programs to Minimize Resistance

### Steps to Minimize Antimicrobial-Drug Resistance and Its Economic Impact

Several strategies and approaches have attempted to deal with resistance (Table 4) (30,31). The term "control" seems inappropriate because true control of antimicrobial-resistant organisms and their effects seems biologically and historically impossible. However, statements from professional societies, independent review groups, and governmental agencies stress several measures to minimize the detrimental effects of resistance (32-35). These include professional educational programs, enhanced microbiologic surveillance, enhanced surveillance of patients, implementation of infection control procedures, development of vaccines against resistant organisms, and prudent use of antimicrobial agents for treatment and prophylaxis. These measures can be evaluated in terms of their success in reducing antimicrobial-drug resistance and its associated costs (36). However, costs associated with each of the strategies must also be included in the calculation of overall economic impact (26). These costs are more or less important, depending on the perspective from which the analysis is being conducted. The few analyses of this type conducted to date focus on costs of infection control (37).

#### Developing New Antimicrobial Drugs and Other Therapeutic Agents

The most obvious way to combat resistance is to develop new antimicrobial agents (38). Several new combinations or classes of antimicrobial agents now may prove valuable to combat infections caused by resistant bacteria (39,40). Nonantimicrobial means to combat resistant organisms (e.g., development of vaccines) will also assume more importance (41,42).

Economic impact here is primarily a concern for the pharmaceutical industry and consists of the net difference between costs associated with developing new agents and the profit from sale of the agents when they are marketed.

#### Surveillance for Antimicrobial-Drug Resistance

Surveillance is vital to determining measures needed to control antimicrobial-drug resistance (43). New, rapid laboratory methods are becoming available to facilitate this important effort. Surveillance methods produce expenses in use of diagnostic testing (e.g., microbiologic cultures), and they require additional time for infection control and laboratory personnel, as well as patient care staff, to interact with infection control personnel and implement surveillance programs.

#### Implementing Infection Control Measures

Approximately 30% to 40% of resistant infections arise from cross-infection via hands of hospital personnel, 20% to

Table 4. Elements of the economic impact of measures to deal with antimicrobial drug resistance, by perspective affected

| Element | Measurement[a] | Perspective affected directly |
|---|---|---|
| Develop new antimicrobial agents | [Costs associated with drug development] - [Profit resulting from new drug's use] | Drug industry, HCB, patient, society |
| Conduct surveillance | [Cost of surveillance for infected and colonized patients (R)] - [Cost of surveillance for infected and colonized patients (S)] | HCB |
| Implement isolation | [Costs associated with barrier isolation (R)] - [Costs associated with barrier isolation (S)] | HCW, visitor, patient, HCB |
| Adapt lab procedures | [Costs associated with testing (R)] - [Costs associated with testing (S)] | HCB, patient, society |
| Educate about resistance | [Costs associated with educational programs (staff, patients) (R)] - [Costs associated with educational programs (staff, patients) (S)] | HCW, patient, visitor, HCB |
| Improve drug administration | [Costs for programs to improve drug administration (R)] - [Costs for programs to improve drug administration (S)] | HCW, HCB |
| Improve drug choice | [Costs for programs to improve drug choice (R)] - [Costs for programs to improve drug choice (S)] | Prescribers, HCB |

[a]R = extent in patients infected with resistant organism; S = extent in patients infected with susceptible organism; HCB = health-care business; HCW = health-care workers.

25% from the selective antimicrobial pressure, 20% to 25% from introduction of new pathogens, and 20% from other or unknown pathways (44). Costs for control of cross-infection include those for masks, gowns, gloves, antiseptics, and other equipment needed for proper isolation precautions; increased personnel time needed to implement isolation procedures; and effort involved in teaching procedures to health-care personnel.

### Adapting Laboratory Methods for Detecting New Types of Antimicrobial-Drug Resistance

Emerging antimicrobial-drug resistance affects the ability of the clinical microbiology laboratory to detect and report resistance. Several new resistance mechanisms in gram-positive and gram-negative bacterial organisms are difficult to detect with usual laboratory methods. To counter these problems, the National Committee for Clinical Laboratory Standards (Villanova, Pennsylvania) and other groups have developed new testing methods, as well as guidelines and standards for testing resistant organisms (45). Costs associated with these efforts are usually borne by the health-care system, whether or not the tests are performed in-house. Patients and society ultimately bear these costs, depending on the mechanism by which the health-care system is paid.

### Educational Programs

Physicians, students, residents, nurses, pharmacists, infection control and quality assurance personnel, administrative staff, and others are frequently part of the health-care team. Making sure that awareness of the problem of antimicrobial-drug resistance and how to deal with it are part of the educational program or in-service education offerings is a key part of obtaining support to minimize resistance. Costs here result from the time needed to prepare and deliver educational presentations and for attendees to participate; these costs are primarily borne by the health-care system.

### Optimizing Antimicrobial Agent Administration

The way that antimicrobial agents are prescribed is a major risk determinant for resistance (46). Programs to monitor and improve procedures for proper dosing, interval of administration, duration of treatment, and monitoring for adverse effects have been undertaken and recently updated (47,48).

The economic impact relates to the time and efforts of prescribers, pharmacists, drug delivery personnel, and administrative staff who provide direct care to patients and set policy in pharmacy and therapeutics committees. Thus, health-care institutions are primarily affected by these attempts to minimize antimicrobial-drug resistance. The combination of measures must be individualized to the particular organism-antimicrobial pair, health-care institution, and specific care setting, for at least two reasons (47). First, the reservoir for important resistant organisms varies dramatically. For some, like MRSA, the reservoir is now in persons in some communities as well as in health-care facilities (49). For others, such as gram-negative bacilli containing extended-spectrum beta-lactamase enzymes, acute-care hospitals (especially intensive care units) and nursing homes are the main reservoir (50). Second, the modes by which different organisms are spread differ. MRSA seems closely linked to person-to-person spread, whereas gram-negative nonfermenting bacilli are often spread through contaminated liquids and respiratory therapy devices. Thus, assessment of economic impact of measures to minimize resistance depends on the specific measures that must be introduced in a given institution or setting.

### Influencing Drug Choice

Recent interest has focused on improving antimicrobial-drug use by controlling the choice of antimicrobial agents by individual prescribers. Some reported efforts attempt to limit use of inappropriate agents by removing specific drugs from the list of available agents in the formulary or restricting them to certain specialists (51,52). Practice guidelines are a means of achieving uniformity of antimicrobial-drug use that have been applied to many areas in addition to that of infectious diseases. Project ICARE (Intensive Care Antimicrobial Drug Resistance Epidemiology) is a cooperative project of the National Nosocomial Infections System of the Centers for Disease Control and Prevention and the Rollins School of Public Health of Emory University. A 1998 survey of 47 hospitals participating in Project ICARE showed that clinical practice guidelines were reported frequently (70% of hospitals) among measures to improve prescribing practices (53). Guidelines are particularly useful in reducing costs of therapy and total costs of prescription, while maintaining quality of care (54). The question is whether these efforts can reduce prevalence of antimicrobial-drug resistance; major successes have been noted in recent studies, both in the community and hospital (54).

### Status of Methods and Results

Measurement of the economic impact of strategies to minimize resistance is imprecise and incomplete (55). Some information is available about the impact of these measures on drug cost and length of hospital stay, number of diagnostic tests, and number of therapeutic drugs used. Further work needed includes designation or identification of optimal methods for measurement, inclusion of more aspects of economic impact, and carefully defining the perspective from which the assessment is being made.

### Conclusions

Determining the true economic impact of antimicrobial-drug resistance is a challenge because so many variables and perspectives are involved. Better methods are needed to assess the practical implications for those from all perspectives, whether prescriber, patient, health-care business, pharmaceutical company, or the public. Because studies completed to date have been hampered by their small size and lack of uniformity, validity of the information provided is unclear and extrapolating the studies to regional or national or international levels is questionable.

Population-based studies of the true impact of resistance would require large multicenter study groups and would be valuable to help address the different perspectives. Relevant studies will require sufficient size to describe baseline antimicrobial-drug resistance, deal with limits of random variation, and control for variables. Multicenter study groups will likely have to be assembled to provide enough observations, as well as sufficient resources. Only when this is done can there be adequate exploration of the true magnitude of the economic impact of antimicrobial-drug resistance.

The economic impact of antimicrobial-drug resistance deserves more attention from government and professional societies. Neither the summary of the Report by the American Society for Microbiology Task Force on Antibiotic Resistance nor the National Coalition on Antibiotic Resistance mentions this as an important area for study or as a concern for health care (32,56). A draft public health action plan to combat antimicrobial-drug resistance published by the federal Interagency Task Force on Antimicrobial Drug Resistance notes that costs of treating resistant infections place a substantial burden on society and mentions the impact of in-hospital cost of six common kinds of resistant bacteria (57).

As the U.S. health-care system has evolved into a business in the past decade, administrators concerned with cost and benefit have become important decision makers. Thus, economic arguments are needed to convince health-system administrators that antimicrobial-drug resistance is a serious issue. The same considerations apply in other countries as well (58). Lack of attention means that funding to solve the problems is unlikely to be found. A change in perception and action is needed to give this important issue of the economic impact of antimicrobial-drug resistance the priority it deserves.

Dr. McGowan is professor of epidemiology and of medicine (infectious diseases) at Emory University. His research interests focus on antimicrobial-drug resistance and its relation to antimicrobial-drug use.

### References

1. Institute of Medicine. Antimicrobial drug resistance: issues and options. Workshop report. Washington: National Academy Press, 1998.
2. Nathwani D, Malek M. Cost considerations in the evaluation of new therapies for gram-positive bacteria. Int J Antimicrob Agents 1999;13:71-8.
3. Scott RD, Solomon SL, McGowan JE Jr. Applying economic principles to health care. Emerg Infect Dis 2000;7(2). In press.
4. Cohen ML. Epidemiology of drug resistance: implications for a post-antimicrobial era. Science 1992;257:1050-5.
5. McGowan JE Jr. Cost and benefit in perioperative antimicrobial prophylaxis-methods for economic analysis. Rev Infect Dis 1991;13(Suppl 10):S879-S889.
6. Coast J, Smith RD, Millar MR. Superbugs: should antimicrobial drug resistance be included as a cost in economic evaluation? Health Economics 1996;5:217-26.
7. Zanetti G, Platt R. Cost-effectiveness of vancomycin versus cefazolin for perioperative antibiotic prophylaxis in coronary artery bypass graft surgery (abstract). Am J Infect Control 2000;28:79.
8. Chrischilles EA, Scholz DA. Dollars and sense: a practical guide to cost analysis for hospital epidemiology and infection control. Clinical Performance and Quality Health Care 1999;7:107-11.
9. Soriano A, Martinez JA, Mensa J, Marco F, Almela M, Moreno-Martinez A, et al. Pathogenic significance of methicillin resistance for patients with *Staphylococcus aureus* bacteremia. Clin Infect Dis 2000;30:368-73.
10. Roghmann M, Bradham D, South B, Fridkin S, Perl TM. The clinical and economic impact of antimicrobial drug resistance on nosocomial bloodstream infections (abstract). Infect Control Hosp Epidemiol 2000;21:97.
11. Vanhems P, Lepape A, Savey A, Jambou P, Fabry J. Nosocomial pulmonary infection by antimicrobial-resistant bacteria of patients hospitalized in intensive care units: risk factors and survival. J Hosp Infect 2000;45:98-106.
12. Simor AE, Kim T, Oh PI. The economic impact of methicillin-resistant *Staphylococcus aureus* in Canadian hospitals (abstract). Infect Control Hosp Epidemiol 2000;21:124.
13. Harthug S, Eide GE, Langeland N. Nosocomial outbreak of ampicillin resistant *Enterococcus faecium*: risk factors for infection and fatal outcome. J Hosp Infect 2000;45:135-44.
14. Bhavnani SM, Drake JA, Forrest A, Deinhart JA, Jones RN, Biedenbach DJ, et al. A nationwide, multicenter case-control study comparing risk factors, treatment and outcome for vancomycin-resistant and -susceptible enterococcal bacteremia. Diagn Microbiol Infect Dis 2000;36:145-58.
15. Feikin DR, Schuchat A, Kolczak M, Barrett NL, Harrison LH, Lefkowitz L, et al. Mortality from invasive pneumococcal pneumonia in the era of antibiotic resistance, 1995-1997. Am J Public Health 2000;90:223-9.
16. Garbutt JM, Ventrapragada M, Littenberg B, Mundy LM. Association between resistance to vancomycin and death in cases of *Enterococcus faecium* bacteremia. Clin Infect Dis 2000;30:466-72.
17. Carmeli Y, Troillet N, Karchmer AW, Samore MH. Health and economic outcomes of antibiotic resistance in *Pseudomonas aeruginosa*. Arch Intern Med 1999;159:1127-32.
18. Rubin RJ, Harrington CA, Poon A, Dietrich K, Greene JA, Moiduddin A. The economic impact of *Staphylococcus aureus* infection in New York City hospitals. Emerg Infect Dis 1999;5:9-17.
19. Weingarten CM, Rybak MJ, Jahns BE, Stevenson JG, Brown WJ, Levine DP. Evaluation of *Acinetobacter baumanii* infection and colonization and antimicrobial treatment patterns in an urban teaching hospital. Pharmacotherapy 1999;19:1080-5.
20. Gonzalez C, Rubio M, Romero-Vivas J, Gonzalez M, Picazo JJ. Bacteremic pneumonia due to *Staphylococcus aureus*: a comparison of disease caused by methicillin-resistant and methicillin-susceptible organisms. Clin Infect Dis 1999;29:1171-7.
21. Abramson MA, Sexton DJ. Nosocomial methicillin-resistant and methicillin-susceptible *Staphylococcus aureus* primary bacteremia: at what costs? Infect Control Hosp Epidemiol 1999;20:408-11.
22. Einarsson S, Kristjansson M, Kristinsson KG, Kjartansson G, Jonsson S. Pneumonia caused by penicillin-non-susceptible and penicillin-susceptible pneumococci in adults: A case-control study. Scand J Infect Dis 1998;30:253-6.
23. Ibelings MM, Bruining HA. Methicillin-resistant *Staphylococcus aureus*: acquisition and risk of death in patients in the intensive care unit. Eur J Surg 1998;164:411-8.
24. Eandi M, Zara GP. Economic impact of resistance in the community. Internat J Clin Pract 1998;95(Suppl):27-38.
25. Bryce EA, Tiffin SM, Isaac-Renton JL, Wright CJ. Evidence of delays in transferring patients with methicillin-resistant *Staphylococcus aureus* or vancomycin-resistant *Enterococcus* to long-term-care facilities. Infect Control Hosp Epidemiol 2000;21:270-1.
26. Liss RH, Batchelor FR. Economic evaluations of antibiotic use and resistance-a perspective: report of Task Force 6. Rev Infect Dis 1987;9(Suppl 3):S297-S312.
27. Harris JD, Samore M, Carmeli Y. Control group selection is an important but neglected issue in studies of antibiotic resistance. Ann Intern Med 2000;133:159.
28. Rennie D, Luft HS. Pharmacoeconomic analyses - making them transparent, making them credible. JAMA 2000;283:2158-60.
29. Neumann PJ, Stone PW, Chapman RH, Sandberg EA, Bell CM. The quality of reporting in published cost-utility analyses, 1976-1997. Ann Intern Med 2000;132:964-72.
30. McGowan JE Jr. Ways and means to influence antimicrobial prescribing in healthcare and its impact on resistance. In: Andremont A, Brun-Buisson C, McGowan JE Jr., editors. Antibiotic therapy and control of antimicrobial drug resistance in hospitals: 6th Maurice Rapin Colloquia. Paris: Elsevier; 1999. p.97-105.
31. McGowan JE Jr. Robert W. Philip Memorial Lecture: Year 2000 bugs--the end of the antibiotic era? Bulletin of the Royal College of Physicians of Edinburgh. In press.

32. American Society for Microbiology. Report of the ASM Task Force on Antibiotic Resistance. Antimicrob Agents Chemother 1995;39(5 Suppl):1-23.
33. Schlaes D, Gerding D, Tenover F, McGowan JE Jr, Levy S, John J. Guidelines for the prevention of antimicrobial drug resistance in hospitals: joint statement by the Society for Health Care Epidemiology of America and the Infectious Diseases Society of America. Infect Control Hosp Epidemiol 1997;18:275-91.
34. Select Committee on Science and Technology, House of Lords. Seventh Report: Resistance to antibiotics and other antimicrobial agents. London: Her Majesty's Stationery Office;1998. Available at URL: http://www.parliament.the-stationery-office.co.uk/pa/ld199798/ldselect/ldsctech/081vii/st0701.htm
35. Department of Health UK. Government Response to the House of Lords Select Committee on Science & Technology Report: Resistance to antibiotics and other antimicrobial agents (publication CM4172). London: The Stationery Office; 1998.
36. McGowan JE Jr. Do intensive hospital antibiotic control programs prevent the spread of antibiotic resistance? Infect Control Hosp Epidemiol 1994;15:478-83.
37. Lai KK, Kelley AL, Melvin ZS, Belliveau PP, Fontecchio SA. Failure to eradicate vancomycin-resistant enterococci in a university hospital and the cost of barrier precautions. Infect Control Hosp Epidemiol 1998;19:647-52.
38. Lavin BS. Antibiotic cycling and marketing into the 21st century: a perspective from the pharmaceutical industry. Infect Control Hosp Epidemiol 2000;21(Suppl):S32-S35.
39. Moellering RC Jr. A novel antimicrobial agent joins the battle against resistant bacteria. Ann Intern Med 1999;130:155-7.
40. Medical Letter. Gatifloxacin and moxifloxacin: two new fluoroquinolones. Med Lett Drugs Ther 2000;42:15-7.
41. Soriano-Gabarro M, Besser R, Schuchat A. Indications for pneumococcal vaccine in the era of expanding pneumococcal resistance. Journal of Critical Illness 2000;15:161-4.
42. Dagan R, Givon-Lavi N, Shkolnik L, Yagupsky P, Fraser D. Acute otitis media caused by antibiotic-resistant *Streptococcus pneumoniae* in southern Israel: implication for immunizing with conjugate vaccines. J Infect Dis 2000;181:1322-9.
43. Wise R, Andrews JM. Local surveillance of antimicrobial drug resistance. Lancet 1998;352:657.
44. Weinstein RA. Controlling antimicrobial drug resistance: the role of infection control and antimicrobial use. Program of the 4th Decennial International Conference on Nosocomial and Health-care-Associated Infections. Atlanta, Georgia, March 5-9, 2000:7.
45. National Committee for Clinical Laboratory Standards. Performance Standards for Antimicrobial Susceptibility Testing: Tenth Informational Supplement (Publication M100-S10). Villanova, Pennsylvania: NCCLS; 2000. vol. 19.
46. Austin DJ, Kristinnson KG, Anderson RM. The relationship between the volume of antimicrobial consumption in human communities and the frequency of resistance. Proc Natl Acad Sci U S A 1999;96:1152-6.
47. McGowan JE Jr. Drug resistance and nosocomial infections: epidemiology and prevention strategies. In: Finch RG, Williams R, editors. Balliere's clinical infectious diseases. London: Balliere Tindall; 1999. p. 177-92.
48. Schentag JJ. Antibiotic dosing—does one size fit all? JAMA 1998;279:159-60.
49. Fraise AP. Guidelines for the control of methicillin-resistant *Staphylococcus aureus*. J Antimicrob Agents Chemother 1998;42:287-9.
50. Jacoby GA. Editorial response: epidemiology of extended-spectrum beta-lactamases. Clin Infect Dis 1998;27:81-3.
51. White AC Jr, Atmar RL, Wilson J, Cate TR, Stager CE, Greenberg SB. Effects of requiring prior authorization for selected antimicrobials; expenditures, susceptibilities, and clinical outcomes. Clin Infect Dis 1997;25:230-9.
52. Burke JP. Antibiotic resistance—squeezing the balloon? JAMA 1998;280:1270-1.
53. Lawton RM, Fridkin SK, Gaynes RP, McGowan JE Jr, ICARE Hospitals. Practices to improve antimicrobial use at 47 US hospitals: the status of the 1997 SHEA/IDSA position paper recommendations. Infect Control Hosp Epidemiol 2000;21:256-9.
54. Gould IM. A review of the role of antibiotic policies in the control of antibiotic resistance. J Antimicrob Chemother 1999;43:459-65.
55. Phelps CE. Bug/drug resistance: sometimes less is more. Med Care 1989;27:194-203.
56. Gerding DN, Martone WJ. SHEA conference on antimicrobial drug resistance. Infect Control Hosp Epidemiol 2000;21:347-51.
57. Interagency Task Force on antimicrobial drug resistance. Draft public health action plan to combat antimicrobial drug resistance. Part I: domestic issues. Available at website: http://www.cdc.gov/drugresistance/actionplan/index.htm. Atlanta: Centers for Disease Control and Prevention; 2000.
58. Coast J, Smith RD, Millar MR. An economic perspective on policy to reduce antimicrobial drug resistance. Soc Sci Med 1998;46:29-38.

## References

[PDF_00633] Abramson M. A., Sexton D. J. (1999). Nosocomial methicillin-resistant and methicillin-susceptible Staphylococcus aureus primary bacteremia: at what costs?. Infect Control Hosp Epidemiol.

[PDF_00718] Austin D. J., Kristinsson K. G., Anderson R. M. (1999). The relationship between the volume of antimicrobial consumption in human communities and the frequency of resistance.. Proc Natl Acad Sci U S A.

[PDF_00607] Bhavnani S. M., Drake J. A., Forrest A., Deinhart J. A., Jones R. N., Biedenbach D. J., Ballow C. H. (2000). A nationwide, multicenter, case-control study comparing risk factors, treatment, and outcome for vancomycin-resistant and -susceptible enterococcal bacteremia.. Diagn Microbiol Infect Dis.

[PDF_00645] Bryce E. A., Tiffin S. M., Isaac-Renton J. L., Wright C. J. (2000). Evidence of delays in transferring patients with methicillin-resistant Staphylococcus aureus or vancomycin-resistant Enterococcus to long-term-care facilities.. Infect Control Hosp Epidemiol.

[PDF_00736] Burke J. P. (1998). Antibiotic resistance--squeezing the balloon?. JAMA.

[PDF_00619] Carmeli Y., Troillet N., Karchmer A. W., Samore M. H. (1999). Health and economic outcomes of antibiotic resistance in Pseudomonas aeruginosa.. Arch Intern Med.

[PDF_00586] Chrischilles E. A., Scholz D. A. (1999). Dollars and sense: a practical guide to cost analysis for hospital epidemiology and infection control.. Clin Perform Qual Health Care.

[PDF_00753] Coast J., Smith R. D., Millar M. R. (1998). An economic perspective on policy to reduce antimicrobial resistance.. Soc Sci Med.

[PDF_00580] Coast J., Smith R. D., Millar M. R. (1996). Superbugs: should antimicrobial resistance be included as a cost in economic evaluation?. Health Econ.

[PDF_00575] Cohen M. L. (1992). Epidemiology of drug resistance: implications for a post-antimicrobial era.. Science.

[PDF_00704] Dagan R., Givon-Lavi N., Shkolnik L., Yagupsky P., Fraser D. (2000). Acute otitis media caused by antibiotic-resistant Streptococcus pneumoniae in southern Israel: implication for immunizing with conjugate vaccines.. J Infect Dis.

[PDF_00643] Eandi M., Zara G. P. (1998). Economic impact of resistance in the community.. Int J Clin Pract Suppl.

[PDF_00636] Einarsson S., Kristjansson M., Kristinsson K. G., Kjartansson G., Jonsson S. (1998). Pneumonia caused by penicillin-non-susceptible and penicillin-susceptible pneumococci in adults: a case-control study.. Scand J Infect Dis.

[PDF_00612] Feikin D. R., Schuchat A., Kolczak M., Barrett N. L., Harrison L. H., Lefkowitz L., McGeer A., Farley M. M., Vugia D. J., Lexau C. (2000). Mortality from invasive pneumococcal pneumonia in the era of antibiotic resistance, 1995-1997.. Am J Public Health.

[PDF_00728] Fraise A. P. (1998). Guidelines for the control of methicillin-resistant Staphylococcus aureus.. J Antimicrob Chemother.

[PDF_00616] Garbutt J. M., Ventrapragada M., Littenberg B., Mundy L. M. (2000). Association between resistance to vancomycin and death in cases of Enterococcus faecium bacteremia.. Clin Infect Dis.

[PDF_00746] Gerding D. N., Martone W. J. (2000). SHEA conference on antimicrobial resistance. Society for Healthcare Epidemiology of America.. Infect Control Hosp Epidemiol.

[PDF_00629] González C., Rubio M., Romero-Vivas J., González M., Picazo J. J. (1999). Bacteremic pneumonia due to Staphylococcus aureus: A comparison of disease caused by methicillin-resistant and methicillin-susceptible organisms.. Clin Infect Dis.

[PDF_00742] Gould I. M. (1999). A review of the role of antibiotic policies in the control of antibiotic resistance.. J Antimicrob Chemother.

[PDF_00652] Harris A. D., Samore M. H., Carmeli Y. (2000). Control group selection is an important but neglected issue in studies of antibiotic resistance.. Ann Intern Med.

[PDF_00604] Harthug S., Eide G. E., Langeland N. (2000). Nosocomial outbreak of ampicillin resistant Enterococcus faecium: risk factors for infection and fatal outcome.. J Hosp Infect.

[PDF_00640] Ibelings M. M., Bruining H. A. (1998). Methicillin-resistant Staphylococcus aureus: acquisition and risk of death in patients in the intensive care unit.. Eur J Surg.

[PDF_00730] Jacoby G. A. (1998). Epidemiology of extended-spectrum beta-lactamases.. Clin Infect Dis.

[PDF_00690] Lai K. K., Kelley A. L., Melvin Z. S., Belliveau P. P., Fontecchio S. A. (1998). Failure to eradicate vancomycin-resistant enterococci in a university hospital and the cost of barrier precautions.. Infect Control Hosp Epidemiol.

[PDF_00694] Lavin B. S. (2000). Antibiotic cycling and marketing into the 21st century: a perspective from the pharmaceutical industry.. Infect Control Hosp Epidemiol.

[PDF_00738] Lawton R. M., Fridkin S. K., Gaynes R. P., McGowan J. E. (2000). Practices to improve antimicrobial use at 47 US hospitals: the status of the 1997 SHEA/IDSA position paper recommendations. Society for Healthcare Epidemiology of America/Infectious Diseases Society of America.. Infect Control Hosp Epidemiol.

[PDF_00649] Liss R. H., Batchelor F. R. (1987). Economic evaluations of antibiotic use and resistance--a perspective: report of Task Force 6.. Rev Infect Dis.

[PDF_00577] McGowan J. E. (1991). Cost and benefit of perioperative antimicrobial prophylaxis: methods for economic analysis.. Rev Infect Dis.

[PDF_00687] McGowan J. E. (1994). Do intensive hospital antibiotic control programs prevent the spread of antibiotic resistance?. Infect Control Hosp Epidemiol.

[PDF_00697] Moellering R. C. (1999). A novel antimicrobial agent joins the battle against resistant bacteria.. Ann Intern Med.

[PDF_00570] Nathwani D., Malek M. (1999). Cost considerations in the evaluation of new therapies for gram-positive bacteria.. Int J Antimicrob Agents.

[PDF_00657] Neumann P. J., Stone P. W., Chapman R. H., Sandberg E. A., Bell C. M. (2000). The quality of reporting in published cost-utility analyses, 1976-1997.. Ann Intern Med.

[PDF_00744] Phelps C. E. (1989). Bug/drug resistance. Sometimes less is more.. Med Care.

[PDF_00655] Rennie D., Luft H. S. (2000). Pharmacoeconomic analyses: making them transparent, making them credible.. JAMA.

[PDF_00622] Rubin R. J., Harrington C. A., Poon A., Dietrich K., Greene J. A., Moiduddin A. (1999). The economic impact of Staphylococcus aureus infection in New York City hospitals.. Emerg Infect Dis.

[PDF_00726] Schentag J. J. (1998). Antibiotic dosing--does one size fit all?. JAMA.

[PDF_00673] Shlaes D. M., Gerding D. N., John J. F., Craig W. A., Bornstein D. L., Duncan R. A., Eckman M. R., Farrer W. E., Greene W. H., Lorian V. (1997). Society for Healthcare Epidemiology of America and Infectious Diseases Society of America Joint Committee on the Prevention of Antimicrobial Resistance: guidelines for the prevention of antimicrobial resistance in hospitals.. Infect Control Hosp Epidemiol.

[PDF_00589] Soriano A., Martínez J. A., Mensa J., Marco F., Almela M., Moreno-Martínez A., Sánchez F., Muñoz I., Jiménez de Anta M. T., Soriano E. (2000). Pathogenic significance of methicillin resistance for patients with Staphylococcus aureus bacteremia.. Clin Infect Dis.

[PDF_00597] Vanhems P., Lepape A., Savey A., Jambou P., Fabry J. (2000). Nosocomial pulmonary infection by antimicrobial-resistant bacteria of patients hospitalized in intensive care units: risk factors and survival.. J Hosp Infect.

[PDF_00625] Weingarten C. M., Rybak M. J., Jahns B. E., Stevenson J. G., Brown W. J., Levine D. P. (1999). Evaluation of Acinetobacter baumannii infection and colonization, and antimicrobial treatment patterns in an urban teaching hospital.. Pharmacotherapy.

[PDF_00732] White A. C., Atmar R. L., Wilson J., Cate T. R., Stager C. E., Greenberg S. B. (1997). Effects of requiring prior authorization for selected antimicrobials: expenditures, susceptibilities, and clinical outcomes.. Clin Infect Dis.

[PDF_00708] Wise R., Andrews J. M. (1998). Local surveillance of antimicrobial resistance. Synercid Resistance Surveillance Group.. Lancet.

